# Retraction Note: Efficacy and immunogenicity of MultiTEP-based DNA vaccines targeting human α-synuclein: prelude for IND enabling studies

**DOI:** 10.1038/s41541-025-01102-3

**Published:** 2025-03-12

**Authors:** Changyoun Kim, Armine Hovakimyan, Karen Zagorski, Tatevik Antonyan, Irina Petrushina, Hayk Davtyan, Gor Chailyan, Jonathan Hasselmann, Michiyo Iba, Anthony Adame, Edward Rockenstein, Marcell Szabo, Mathew Blurton-Jones, David H. Cribbs, Anahit Ghochikyan, Eliezer Masliah, Michael G. Agadjanyan

**Affiliations:** 1 Laboratory of Neurogenetics, National Institute of Aging, National Institute of Health, Bethesda, MD USA; 2 Department of Molecular Immunology, Institute for Molecular Medicine, Huntington Beach, CA USA; 3 Institute for Memory Impairments and Neurological Disorders, University of California Irvine, Irvine, CA USA; 4 Sue and Bill Gross Stem Cell Research Center, University of California Irvine, Irvine, CA USA; 5 Department of Neurosciences, University of California, San Diego, La Jolla, CA USA

**Keywords:** Parkinson's disease, DNA vaccines

**Retraction to:** ***npj Vaccines*** 10.1038/s41541-021-00424-2, **published online 10 January 2022**

The Editor-in-Chief has retracted this article because of concerns regarding figures presented in this work. These concerns call into question the article’s overall scientific soundness. An investigation conducted after its publication discovered that panel α-synuclein TG PV-1950D, Male Hippocamp in Figure 7a appears to overlap, when rotated, with panel α-synuclein TG PV-1950D, Female Hippocamp in Figure 7c. These panels represent tissues taken from different animals. The Authors provided some raw data on request from the Publisher. However, this was insufficient to satisfy the concerns raised. In light of previous concerns involving figures presented in this article, the Editor-in-Chief therefore no longer has confidence in the integrity of the research presented in this article.

Michael G. Agadjanyan has stated on behalf of Armine Hovakimyan, Karen Zagorski, Tatevik Antonyan, Gor Chailyan, Anahit Ghochikyan, Irina Petrushina, Mathew Blurton-Jones, David H. Cribbs, and Hayk Davtyan that they disagree with the retraction. Eliezer Masliah disagrees with the retraction. Jonathan Hasselmann agrees with the retraction. The Publisher was unable to contact Anthony Adame. Changyoun Kim, Michiyo Iba, and Marcell Szabo have not replied to correspondence from the Publisher. The Publisher has been informed that Edward Rockenstein has passed away.

